# RCC Real-World Data: Prognostic Factors and Risk Stratification in the Immunotherapy Era

**DOI:** 10.3390/cancers14133127

**Published:** 2022-06-26

**Authors:** Shira Sagie, Michal Sarfaty, Meital Levartovsky, Hadas Gantz Sorotsky, Raanan Berger, Ruth Percik, Moran Gadot

**Affiliations:** 1 Institute of Oncology, Sheba Medical Center, Ramat Gan 52621, Israel; shirasagie@gmail.com (S.S.); michalchen1@gmail.com (M.S.); meital.levartovsky@sheba.health.gov.il (M.L.); hadas.gantzsorotsky@sheba.health.gov.il (H.G.S.); raanan.berger@sheba.health.gov.il (R.B.); ruth.percik@sheba.health.gov.il (R.P.); 2 Sackler Faculty of Medicine, Tel Aviv University, Tel Aviv 6997801, Israel; 3 The Sheba Talpiot Medical Leadership Program, Sheba Medical Center, Ramat Gan 52621, Israel; 4 Division of Endocrinology, Diabetes and Metabolism, Sheba Medical Center, Ramat Gan 52621, Israel

**Keywords:** checkpoint inhibitors, renal cell carcinoma, risk prognostication

## Abstract

**Simple Summary:**

Nowadays, most metastatic renal cell carcinoma (mRCC) patients are candidates for immunotherapy. Risk stratification is based on the IMDC model that was developed in an earlier era of vascular endothelial growth factor receptor inhibitors (VEGFRi). An updated, more accurate model is needed. This paper suggests an updated risk-stratification model based on five factors that are strongly correlated with overall survival in a real-world cohort of patients with mRCC treated with checkpoint inhibitors in any line of treatment during the course of their disease. Compared with the commonly used IMDC criteria, in this cohort, our model was better able to predict survival.

**Abstract:**

Immunotherapy has transformed the landscape of treatment in metastatic renal cell carcinoma (mRCC) in the last decade. Currently, prognostic risk stratification is based on the model developed in the era of vascular endothelial growth factor receptor inhibitors (VEGFRi) by Heng in 2009. Our study aims to find the most relevant risk criteria for mRCC patients treated with checkpoint inhibitors (CPI). In a retrospective cohort study, laboratory, pathology, demographic, and clinical data were retrieved from electronic medical records of consecutive mRCC patients treated with CPI in a tertiary center between 2015 and 2020. An unbiased multivariate analysis was performed to define predictive variables with a bootstrap validation step. We analyzed data on 127 patients with a median follow-up of 60 months. The median overall survival (OS) since the diagnosis of metastatic disease was 57 months. The response rate for CPI was 39%. Five risk factors were correlated with worse OS: intact primary kidney tumor (HR 2.33, *p* = 0.012), liver metastasis (HR 3.33, *p* = 0.001), <one year to treatment start (HR 1.98, *p* = 0.029), elevated platelets (HR 3.06, *p* = 0.015), and Karnofsky performance status <80% (HR = 3.42, *p* = 0.001). The model received a C-index of 70.7 compared with a score of 62.0 for the Heng’s model. When dividing patients into “low-risk” (0–1 risk factors) and “high-risk” (2–5 risk factors), there was good separation between the groups, with an HR of 5.9 (*p* < 0.0001). This study presents a new prognostic model for mRCC in the immunotherapy era with improved accuracy. Further research is needed to validate this model in larger cohorts.

## 1. Introduction

Metastatic renal cell carcinoma (mRCC) is characterized by a diverse clinical manifestation, ranging from an asymptomatic disease with a long life expectancy to a tumultuous disease with a dismal prognosis despite oncological treatment [1]. Over the years, clinical and laboratory characteristics representing the biology of the tumor have been found to correlate with patient survival. Combinations of these characteristics made it possible to produce survival prediction models.

Motzer et al. published the first version of the Memorial Sloan-Kettering Cancer Center (MSKCC) prognostic model for mRCC in 1991, which divided patients into three risk groups according to their overall survival by the presence or absence of five risk factors, including Karnofsky performance status (KPS), serum lactate dehydrogenase, hemoglobin, calcium, and the absence of prior nephrectomy [2]. This model was developed during the cytokine era, when cytokine interleukin 2 (IL-2) and interferon alfa (IFNa) were common treatments for mRCC [3,4,5,6,7] and the standard stratification tool for all major phase III trials, leading to the registration of approved targeted agents in the 2000s. This model was validated in 2005 and has been extended to also include prior radiotherapy and sites of metastasis [8]. Other prognostic models also include the number of metastatic sites [9].

Sunitinib and Pazopanib, which are vascular endothelial growth factor receptor inhibitors (VEGFRi), were then used as a first-line treatment for mRCC, as they proved to be superior to cytokines in terms of overall survival (OS), progression-free survival (PFS), response rate (RR), and quality of life (QOL) [10,11,12]. 

Heng et al. presented a new risk-stratification model in 2009 that is based solely on patients treated with VEGFRi; it is also termed the International Metastatic Renal-Cell Carcinoma Database Consortium (IMDC) criteria [13,14]. The model divides patients into favorable, intermediate, and poor risk groups according to six prognostic factors (KPS, time from diagnosis to treatment, hemoglobin, calcium, neutrophil, and platelet count). Additional models have also suggested the number of metastases and alkaline phosphatase as risk factors [15], as well as the presence of bone metastases [16,17], elevated CRP, and the neutrophil-to-lymphocyte ratio [18].

Since 2015, checkpoint inhibitors (CPI) have routinely been used in the treatment of mRCC. Nivolumab was the first PD1 inhibitor to show improved overall survival after VEGFRi compared with everolimus [19]. A recent systematic review of mRCC patients who received second-line treatment described clinical-pathological features correlated to the response to nivolumab, including PS and the number of metastatic sites but not prior nephrectomy [20]. Prospective real-world data on the safety and efficacy of nivolumab in second-line RCC demonstrated the correlation between the number of IMDC risk factors to OS [21].

By the end of 2017, combinations of immunotherapy replaced VEGFRi in the first line of treatment for intermediate- and poor-risk patients since ipilimumab plus nivolumab showed improved OS compared with sunitinib [22]. Four immuno-VEGFRi combinations also showed benefit over sunitinib: avelumab plus axitinib [23], pembrolizumab plus axitinib [24], nivolumab plus cabozantinib [25], and pembrolizumab plus lenvatinib [26].

The combination therapies mentioned above were not directly compared; however, Heng’s risk prognostication model has been used for stratification in all of these phase III trials and has proven effective in predicting the superiority of immunotherapy combinations versus Sunitinib in intermediate–poor-risk patients in terms of PFS and OS. Currently, all evidence-based recommendations for systemic treatment are based on Heng’s risk group stratification, which was built and analyzed in a previous era of treatment. Moreover, patient-specific molecular and clinical data suggest that patterns of response and resistance to VEGFRi and immunotherapy are innately different and depend on tumor biology [27,28].

Nowadays, when most patients are candidates for immunotherapy throughout the course of their metastatic disease, we set out to identify, in an unbiased manner, the clinical risk factors associated with prognosis under immunotherapy and compare them to the known risk factors in the Heng model, which was developed in the era of VEGFRi.

## 2. Materials and Methods

Study population and design: We conducted this study using a retrospective cohort study of consecutive patients treated with immunotherapy at Sheba Medical Center between January 2015 and December 2020. All patients treated for mRCC with CPI were included if they received nivolumab, pembrolizumab, or avelumab as monotherapy or in combination therapy, for any line of treatment, as a standard of care or in a clinical trial (*n* = 127). Patient demographics, tumor details, and treatment and outcome data were retrieved from patients’ electronic records.

Study variables: We collected patients’ demographics, including sex, age, smoking status, and background autoimmune diseases; baseline RCC characteristics, including date of initial diagnosis and of metastatic diagnosis, nephrectomy status, RCC histology, presence of sarcomatoid features, metastasis number and sites at diagnosis of metastatic disease, presence of neutrophilia, thrombophilia, anemia, hypercalcemia, less than one year between diagnosis and treatment initiation (time criteria), and ECOG performance status (PS); and treatment details, including the type of CPI, previous and subsequent VEGFRi, and immune-related adverse events (irAE) on CPI. Overall survival (OS) was defined since the diagnosis of metastatic disease.

Statistical analysis: The study sample was described using medians and interquartile range (IQR) for variables with skewed distribution, frequencies, and percentages for categorical variables. Cox proportional hazard regression models were used to estimate hazard ratios (HR) with 95% confidence intervals (CIs) for OS for both univariate and multivariate models. Correlations between the independent variables were analyzed by Spearman’s correlation coefficients prior to their inclusion in the multivariable models. Variables with a correlation coefficient of 0.6 or above were studied in separate models. We fitted multivariable Cox proportional hazards models in a stepwise manner, and a significance level of 0.2 was used for entering and removing variables. We compared models by their concordance index (C-index). In order to assess the internal validity of our multivariable model, we bootstrapped 100 samples of 75% of our data and rebuilt multivariate models stepwise. We calculated the frequency of each variable that was included in the resulting models from the 100 bootstrap samples. Risk factors that were present in more than half of the models were considered significant. Next, we analyzed the coefficients of each model and compared our original model coefficients with the mean coefficients of all models built from bootstrap samples. Kaplan–Meier curves with a log-rank test were used to compare OS between new risk groups. *p* < 0.05 was considered statistically significant. Median follow-up time was calculated by the reversed Kaplan–Meier method. Missing data: we were able to obtain 98% of all data points, and the final model was built on 122 patients. The statistical analyses were performed using R version 3.4.2.

## 3. Results

The characteristics of the baseline patients and treatments are summarized in Table 1. Overall, 127 patients were included. The median age at metastatic disease diagnosis and CPI initiation was 62 and 64 years, respectively. The majority of patients were male (72%). Clear cell histology was identified in 87% of patients. Nearly 80% of patients had prior nephrectomy. The lungs were the most common site of metastases (65%). Liver and brain metastases were present in 13% and 5.5% of cases, respectively, in terms of the time of metastatic disease presentation. Most of the patients were considered intermediate-risk by the Heng criteria (66%). CPI was given in the first line in 46.5%, in the second line in 40.2%, and in the third line or more in 13.4%. Patients were treated with a CPI combination (Ipilimumab plus Nivolumab) in 48% of cases and CPI-VEGFRi combination in 19.7%, and the rest of the patients received single-agent CPI.

**Figure 1:**
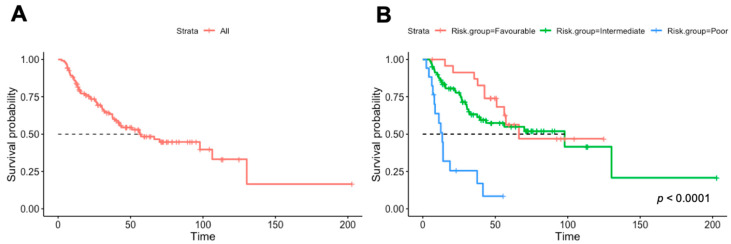
Kaplan–Meier plots of overall survival of all patients (**A**) and by Heng risk groups (**B**). *p*-value calculated by a Cox proportional hazard regression model.

The median follow-up was 60 months. At the time of data analyses, 61 (48%) events occurred. In the overall cohort, the median OS was 57 months from metastatic disease diagnosis (Figure 1A) and 33 months from the first immunotherapy treatment. The median OS by Heng criteria was 67.3 months in the favorable-risk group (*n* = 25), 97.8 months in the intermediate-risk group (*n* = 82), and 13.4 months in the poor-risk group (*n* = 17) (Figure 1B). The response rate in our cohort was 39% (9% complete response and 30% partial response), and 16% had a mixed response or stable disease as the best response.

### 3.1. Univariable Analysis

First, we analyzed univariate Cox models for each variable described above and obtained hazard ratios (HR) for survival since metastatic disease diagnosis. The most significant poor prognostic factors were platelets > ULN (HR 5.0, *p* = 0.001), brain metastases (HR 4.24, *p* = 0.019), liver metastases (HR 3.55, *p* < 0.01), and KPS < 80% (HR 3.29, *p* = 0.001). Nephrectomy was a significant protective factor (HR 0.37, *p* = 0.002).

### 3.2. Multivariable Analysis

We performed a stepwise multivariate analysis as described in the methods and validated it with bootstrap analysis. Five variables were highly significant both in the multivariable analysis and in the bootstrap validation process, and two of them were not included in the Heng criteria: intact primary kidney tumor (HR 2.33, *p* = 0.012) and liver metastasis (HR 3.33, *p* = 0.001). The additional three variables appeared in the Heng criteria as well: time criteria (<1 year to treatment start) (HR 1.98, *p* = 0.029), platelets > ULN (HR 3.06, *p* = 0.015), and KPS < 80% (HR = 3.42, *p* = 0.001) (Table 2). Using these five variables, we received a high C-index of 70.7 for this model. In comparison, the Heng criteria model received a C-index of 62.0 in our cohort.

All the above-mentioned variables appeared in more than 80% of the models built on the bootstrap populations; the parameter means of these models appear in Table 2 and were very similar to the HR received on our full study population.

### 3.3. Scoring System

**Figure 2:**
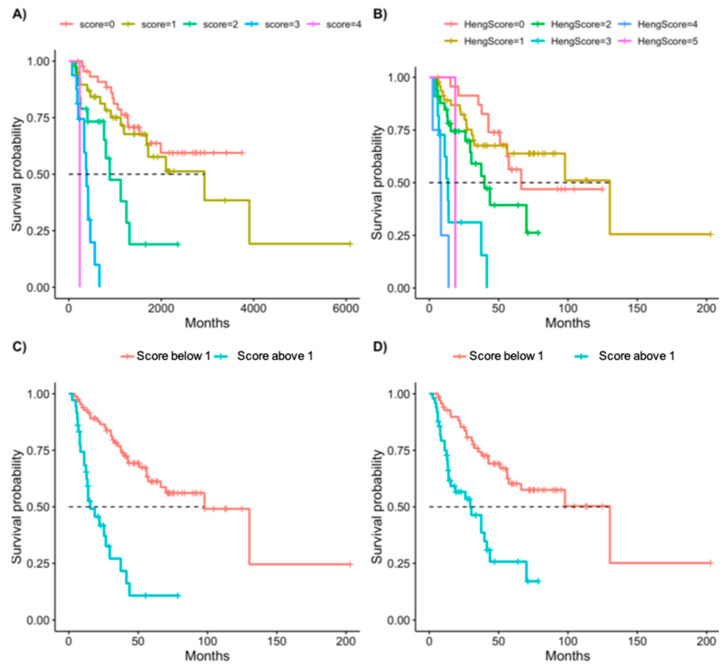
Kaplan–Meier plots of overall survival of patients divided by number of risk factors, by our new model (**A**) and by Heng’s criteria model (**B**). Separation of the patients into two risk groups, high risk (defined by having two or more risk factors) and low risk (fewer than two risk factors) of our new model (**C**) or Heng’s criteria model (**D**).

Survival analysis of our cohort of CPI-treated patients by Heng criteria did not yield a significant difference between intermediate and favorable risk (Table 1 and Figure 1). We suggest a new scoring system, based on the model described above, with each variable receiving one point (intact primary tumor, presence of liver metastases, and less than 1 year between diagnosis and treatment start, KPS < 80%, PLT > ULN). We analyzed Kaplan—Meyer survival curves based on the number of points each patient received (Figure 2A) and compared them to the scoring based on the Heng criteria (Figure 2C). We analyzed the HR of each additional point in each of the models (the new score compared to the Heng criteria) and found a linear trend of the coefficients in our model but not in the Heng score. No patient in our cohort had all risk factors (Table 3). We separated our patients into two risk groups based on our new model: the “low-risk” group included patients with zero to one risk factor, and the “high-risk” group included patients with two or more risk factors (Figure 2B). The HR for the OS of the high-risk group in this classification is 5.9 (*p* < 0.0001). We analyzed the same separation based on the Heng criteria, i.e., patients with two or more risk factors are defined as high risk (Figure 2D), and this Heng high-risk group received an HR of 3.3 (*p* < 0.0001).

## 4. Discussion

Immunotherapy has revolutionized cancer treatment with high response rates and long, durable responses in mRCC [1]. The variety of available life-prolonging CPI-based treatment combinations has made treatment decisions challenging, as these combinations have not been compared to each other. The treatment is chosen to take into account the patient’s clinical condition [29] and the risk group as defined by Heng’s criteria developed in 2009 in a completely different clinical scenario when the main treatment options were VEGFRi.

Our study evaluated the prognostic value of this main clinically utilized risk stratification tool—the Heng criteria—in a modern-era cohort of mRCC patients (2015–2020), all treated with CPI in any treatment line. Our cohort confirms the improved median OS with modern treatments of nearly five years, similar to the reported data in immunotherapy clinical trials [30]. This compares to a median OS of only two years in Heng’s original report. We found that Heng’s intermediate risk and favorable risk group survival plots fail to separate in our cohort.

Molecularly distinct responses to different treatments [26], as well as the longer survival times since Heng criteria analysis, necessitate a new unbiased classification of prognostic risk factors for mRCC.

Herein, we performed an unbiased multivariate analysis of our data to examine a possible new risk model, and we compared it with the historical model. We included clinical, pathological, and laboratory values. Future prognostic models for CPI combination therapies may be enhanced by the inclusion of genetic markers or gene signatures, but these are currently clinically unavailable [29].

Although several of the previous risk factors remained in our new model (KPS, platelets > ULN, time to treatment), two additional factors have entered the model (liver metastasis and intact primary tumor). Other risk factors in the Heng criteria—hypercalcemia, neutrophilia, and anemia—failed to enter the model. In our single-center cohort, the new model exhibits a high concordance index and a linear trend of the coefficients of additional scoring points.

The prognostic value of the liver metastatic site in mRCC is well-known [31,32,33]. The number of metastatic sites at the presentation and metastases in either liver, bone, or central nervous system (CNS) was correlated with worse outcomes in a favorable and intermediate risk cohort of 134 patients, of which 19% were treated with CPI [31]. In an external validation of the above model in a cohort of 1073 patients, of which 22.5% were treated with CPI, the addition of brain, bone, and/or liver metastases to the other Heng variables improved the predictive power of the model. Fifteen percent of patients in this study had a modification of their initial risk category [32]. In a small retrospective cohort (*n* = 37) of patients who received Nivolumab in subsequent lines of therapy, the Heng risk group, in addition to liver and CNS metastases, were associated with worse OS, with an estimated hazard ratio of 4.76 (95% confidence interval (CI), 2.05–19.8) for liver metastases and 2.27 (95% CI, 1.13–28.9) for CNS metastases [33].

The nephrectomy risk factor (termed intact primary tumor in our analysis) was analyzed in the Heng model and demonstrated a significant difference in survival (27-month vs. 11-month *p* < 0.0001) in the univariate analysis but failed to enter their multivariate final model, which may be due to high rate of nephrectomy in their cohort [14]. In our dataset, nephrectomy was a strong protective factor in both univariate and multivariate analyses (HR 2.8 and 2.3 for intact primary tumor in the univariate and multivariate analysis, respectively). Renal cell carcinoma is known in some cases to metastasize many years after the primary tumor was surgically removed. Such dormant metastases may be contained and silenced by the immune system for many years until they return to proliferate [34]. It is plausible that these tumors, which might have long been managed by the immune system, will have a better response and thus better survival in the immunotherapy era.

The majority of mRCC patients are classified as being of intermediate risk (1–2 risk factors), at approximately 52%, according to the Heng model. The intermediate-risk group is heterogeneous in nature, with distinct clinical outcomes under VEGFRi treatment [35]. Many current clinical trials divide their survival analysis into two prespecified groups—favorable- and intermediate + poor-risk groups. We found it more clinically convenient to use our new risk stratification model to divide patients into two groups (high risk/low risk) rather than three. This dichotomous classification (with a cutoff of above one risk factor for high-risk patients) resulted in a strong HR of 5.9 (*p* < 0.0001) for the high-risk group, which includes about 30% of the patient population.

Our cohort included consecutive patients that received immunotherapy in any treatment line. The Heng model was developed on treatment-naïve patients, but it was found to be prognostic for second-line outcomes as well [36]. In an additional study, the Heng criteria appropriately stratified patients into favorable-risk, intermediate-risk, and poor-risk groups for OS, in the second line through the fourth line [37]. Interestingly, the response rate for immunotherapy was not found to deteriorate from the first line to the fourth line.

Although our study analyzed an up-to-date cohort of patients who were all treated with immunotherapy, it has several limitations in the ability to generalize the model to other patient populations. First, it is a retrospective single-center study of the largest cancer center in Israel. Second, due to the recent changes in guidelines and the entrance of new life-prolonging treatments, longer follow-up may be needed to confirm our findings. In addition, as mentioned, patients were included in the study regardless of the treatment line in which they received immunotherapy, although this may not significantly affect the model as described above.

## 5. Conclusions

In summary, our study presents a new prognostic model for mRCC in the immunotherapy era and compares it to the widely used Heng criteria with improved accuracy. Further research in additional patient cohorts and in a homogenous prospective cohort is needed to validate this model.

## Figures and Tables

**Table 1:** Patient characteristics of study population and univariable hazard ratios.

Characteristics	Overall (*n*, %)	Univariable HR (CI, *p*-Value)
Gender, Male (%)	91 (71.7)	0.86 (0.48–1.54, *p* = 0.606)
Age at diagnosis (median [IQR])	62.00 [52.00, 69.00]	1.00 (0.97–1.02, *p* = 0.833)
Smoking, yes (%)	43 (35.0)	1.03 (0.57–1.88, *p* = 0.912)
Sarcomatoid elements, yes (%)	18 (17.6)	1.32 (0.63–2.79, *p* = 0.462)
RCC subtype (%)		
Clear cell	107 (87.0)	
Chromophobe	2 (1.6)	* 1.15 (0.58–2.28, *p* = 0.682)
Papillary	10 (8.1)	
Other	4 (3.2)	
Intact primary kidney tumor (%)	27 (21.3)	2.8 (1.58–4.92, *p* = 0.0004)
Number of metastatic sites (median [IQR])	2.00 [1.00, 3.00]	1.55 (1.18–2.05, *p* = 0.002)
Number of metastatic sites (%)		
1	45 (35.4)	
2	43 (33.9)	
3	25 (19.7)	
4	13 (10.2)	
Lymph node metastases, yes (%)	44 (34.6)	1.93 (1.10–3.38, *p* = 0.022)
Lung metastases, yes (%)	83 (65.4)	0.91 (0.51–1.63, *p* = 0.754)
Bone metastases, yes (%)	41 (32.3)	0.98 (0.54–1.79, *p* = 0.952)
Brain metastases, yes (%)	7 (5.5)	4.24 (1.27–14.12, *p* = 0.019)
Pancreas metastases, yes (%)	7 (5.5)	0.30 (0.04–2.15, *p* = 0.228)
Soft tissue metastases, yes (%)	20 (15.7)	1.55 (0.75–3.18, *p* = 0.237)
Liver metastases, yes (%)	17 (13.4)	3.55 (1.79–7.03, *p* < 0.001)
Adrenal metastases, yes (%)	16 (12.6)	1.15 (0.49–2.69, *p* = 0.754)
Heng risk group (%)		
Favorable	25 (20.0)	
Intermediate	83 (66.4)	1.15 (0.57–2.34, *p* = 0.697)
Poor	17 (13.6)	6.25 (2.69–14.51, *p* < 0.001)
Time criteria, yes (%)	66 (52.8)	2.41 (1.36–4.27, *p* = 0.002)
Performance status criteria, yes (%)	13 (10.5)	3.29 (1.58–6.87, *p* = 0.001)
Hemoglobin criteria, yes (%)	75 (61.0)	1.85 (1.02–3.36, *p* = 0.044)
Neutrophil criteria, yes (%)	8 (6.5)	2.76 (1.08–7.03, *p* = 0.033)
Platelet criteria, yes (%)	10 (8.1)	5.08 (2.02–12.78, *p* = 0.001)
Calcium criteria, yes (%)	3 (2.5)	1.25 (0.17–9.41, *p* = 0.825)
Received VEGFRi before CPI, yes (%)	67 (52.8)	1.14 (0.64–2.03, *p* = 0.661)
Previous treatment lines (median (IQR))	1.00 [0.00, 1.00]	0.90 (0.64–1.28, *p* = 0.565)
Number of treatment lines prior to CPI (%)		
0	59 (46.5)	
1	51 (40.2)	
2	14 (11.0)	
3 or more	3 (2.4)	
CPI response rate (complete and partial) (%)	47 (38.8)	0.2 (0.1–0.4, *p* < 0.001)

RCC—renal cell carcinoma; VEGFRi—vascular endothelial growth factor inhibitor; CPI—checkpoint inhibitor; IQR—interquartile range. * The presented HR is for any histology other than the clear cell compared to clear cell.

**Table 2:** Final multivariable model coefficients and bootstrap estimations.

Variables	Final Multivariable Model				Bootstrap	
	HR	2.5%	97.5%	*p*-Value	Percentage of Times Variable Entered Model	Bootstrap Parameter Means
Intact primary kidney tumor	2.33	1.202	4.502	0.012	94	2.35
Liver metastases	3.33	1.671	6.623	0.001	100	3.27
Time criteria	1.98	1.072	3.641	0.029	86	2.02
Performance status criteria	3.42	1.654	7.077	0.001	100	3.44
Platelet criteria	3.06	1.245	7.514	0.015	85	3.257

**Table 3:** Hazard ratios for our new scoring system compared with the Heng Score.

New Score					Heng Score				
	HR	2.50%	97.50%	*p*-Value		HR	2.50%	97.50%	*p*-Value
One point	1.36	0.67	2.75	0.40	One point	0.94	0.43	2.03	0.87
Two points	4.41	1.99	9.74	0.00	Two points	2.13	0.96	4.70	0.06
Three points	18.17	7.38	44.73	0.00	Three points	7.47	2.96	18.86	0.00
Four points	45.91	5.26	400.49	0.00	Four points	21.64	6.17	75.90	0.00
					Five points	7.49	0.93	60.55	0.06

## Data Availability

Data are available for bona fide researchers who request it from the authors.
